# The Shapley value for a fair division of group discounts for coordinating cooling loads

**DOI:** 10.1371/journal.pone.0227049

**Published:** 2020-01-10

**Authors:** Sasan Maleki, Talal Rahwan, Siddhartha Ghosh, Areej Malibari, Daniyal Alghazzawi, Alex Rogers, Hamid Beigy, Nicholas R. Jennings

**Affiliations:** 1 Department of Computer Engineering, Sharif University of Technology, Tehran, Iran; 2 Computer Science, New York University Abu Dhabi, Abu Dhabi, UAE; 3 Electronics and Computer Science, University of Southampton, Southampton, United Kingdom; 4 Computer Science Department, King Abdulaziz University, Jeddah, Saudi Arabia; 5 Information Systems Department, King Abdulaziz University, Jeddah, Saudi Arabia; 6 Department of Computer Science, Oxford University, Oxford, United Kingdom; 7 Department of Computing, Imperial College London, Oxford, United Kingdom; Shandong University of Science and Technology, CHINA

## Abstract

We consider a demand response program in which a block of apartments receive a discount from their electricity supplier if they ensure that their aggregate load from air conditioning does not exceed a predetermined threshold. The goal of the participants is to obtain the discount, while ensuring that their individual temperature preferences are also satisfied. As such, the apartments need to collectively optimise their use of air conditioning so as to satisfy these constraints and minimise their costs. Given an optimal cooling profile that secures the discount, the problem that the apartments face then is to divide the total discounted cost in a fair way. To achieve this, we take a coalitional game approach and propose the use of the Shapley value from cooperative game theory, which is the normative payoff division mechanism that offers a unique set of desirable fairness properties. However, applying the Shapley value in this setting presents a novel computational challenge. This is because its calculation requires, as input, the cost of every subset of apartments, which means solving an exponential number of collective optimisations, each of which is a computationally intensive problem. To address this, we propose solving the optimisation problem of each subset suboptimally, to allow for acceptable solutions that require less computation. We show that, due to the linearity property of the Shapley value, if suboptimal costs are used rather than optimal ones, the division of the discount will be fair in the following sense: each apartment is fairly “rewarded” for its contribution to the optimal cost and, at the same time, is fairly “penalised” for its contribution to the discrepancy between the suboptimal and the optimal costs. Importantly, this is achieved without requiring the optimal solutions.

## Introduction

The transition to a smart electricity grid presents one of the greatest engineering challenges of this century, as countries face dwindling non-renewable energy sources and work to minimise the adverse effects of greenhouse gas emissions [1]. The Smart Grid represents a modern vision of a dynamic electricity grid, where the delivery, monitoring, and control of power are fully automated [2]. Currently, due to the high costs of upgrading equipment, it is often more economically viable for countries to create smart grids within the limits of the existing infrastructure, and pave way for the integration of renewable sources. However, this places a burden on conventional suppliers with ageing equipment to better manage supply and demand such that not only is the balance maintained at all times, but also peak demand is reduced.

Peaks in consumption are a consequence of unregulated demand. Meeting peaks needs large generation capacities which are only in use during peak periods. This is a significant inefficiency from a technical and financial point of view. This problem can be addressed through demand response programs by distributing peak loads throughout the day. To encourage consumers, financial incentives must be given, so that they shift their loads to off-peak periods. Policies that enforce such user behaviour are based on dynamic pricing which take into account changes in demand. This is in contrast to static pricing, such as flat tariffs, which offer electricity at fixed rates, regardless of the load.

Researchers divide electricity tariffs into the following categories: flat, block rate, seasonal, time of use, superpeak time of use, critical peak, variable peak, real-time, and peak-time rebates [3–5]. Under flat tariffs, prices remain fixed even with changes in demand. Block rate tariffs offer tier based pricing, where higher levels of consumption are charged at higher rates. Seasonal rates change from one season to another, to reflect increase or decrease in demand due to the time of year. Time of use, superpeak time of use, critical peak and variable peak charge users based on pre-declared rates that vary depending on the time of day. They are generally designed such that prices are high during peak hours and low during off-peak hours, with the difference being the duration and start of peak hours. Real-time tariffs offer prices that are adjusted every few minutes to the real cost of generation and delivery. Finally, peak time rebate schemes provide rebates for consuming below a predetermined threshold during peak hours.

Against this background, we design a demand response program whereby a block of apartments are rewarded for coordinating their cooling loads. More specifically, in order to encourage consumers to use less energy for air conditioning, which constitutes a significant amount of electricity consumption in warm-climate countries [6, 7], the electricity supplier offers a discount scheme similar to peak time rebate tariffs, but for a group of apartments. If the aggregate cooling load of all apartments in a block that sign up to the scheme does not exceed a certain threshold, they will be offered a discounted price for the entire day. To achieve the discount, the apartments coordinate and optimise their loads collectively, while also ensuring that their individual temperature preferences are met. Each householder specifies their preference in terms of deviations of the internal temperature from a setpoint temperature over a period of the day during which comfort is desired. Given a coalition of apartments, one can formulate a binary integer program so as to find the optimal cooling plan of each member, such that all temperature constraints as well as the load threshold are all satisfied. A cooling plan determines the periods of time when each apartment is allowed to turn on their air conditioning, which in turn, determines the cost of individual apartments.

Once the cooling plans that secure the discount are determined, the primary question that arises is how the apartments should divide the discount among themselves. Since the apartments need to cooperate together to get the discount, it is important that the way the discount is divided prevents abuse. For instance, it is possible that some apartments act as free-riders [8, 9]. That is, some apartments may not cooperate as much but reap the benefit of the joint effort at the cost of other apartments. Therefore, our aim is to put forward a division mechanism that is fair to all participants in the program. The most straightforward solutions that may come to mind are perhaps an equal division, or one proportional to consumptions. The former is obviously not necessarily fair, and the latter simply overlooks the complex interdependencies between apartments that actually result in the desirable coordination of loads. Moreover, as we discuss later, the thermal characteristics of apartments such as how well they are insulated, and how strict their preferences are, have a direct relationship with their ability to shift their loads. Therefore, the division must ensure that each apartment receives a share of the discount that corresponds to how much they have actually contributed to meeting the threshold and obtaining the discount.

Given this problem and its cooperative nature, we first propose that the apartments in the program are modelled as agents that form a coalition with the common goal of coordinating their cooling loads. This allows us to apply methods from cooperative game theory that will enable us to appropriately distribute the payoff of the game, which we define as the total cost of the grand coalition (i.e., the coalition that consists of all agents).

There are extensive studies in the smart grid domain on using cooperative game theory in this context [10–14]. The majority of payoff distribution concepts in the literature are concerned with some sort of stability of coalitions. A well known such concept is called the *core*, which is a set of distributions that satisfy an exponential number of certain constraints [15]. If a payoff distribution is a member of the core it guarantees that no subgroups of agents can be better off by breaking away and forming a different coalition. The drawback of the core is that, in the general case, it does not always exist and is difficult to compute. In fact, for several well studied problems it is co-NP-complete to even check whether a payoff distribution is a member of the core [16, 17]. Another stability concept, called the bargaining, deals with the dissatisfaction of agents with the share of payoff they receive. Suppose that an agent that is allocated a share of the payoff is unsatisfied with it. It can make an objection to it by requesting some part of the share of a particular agent. If that agent does not challenge this request, it is called a justified objection. The set of payoff distributions in which no agent has a justified objection against any other agent is the bargaining set and is always a superset of the core [18]. A somewhat similar concept is the *kernel* with weaker stability constraints than the core. The idea is that agents compare their strength with one another in terms of how much more they can gain by forming a new coalition. If their strengths differ, stronger agents can threaten to leave, unless they receive a higher payoff that balances the strengths [15].

In addition to the stability solution concepts, the Shapley value is a widely celebrated method in the literature that focuses on fairness of payoff distributions [19]. It satisfies a number of desirable fairness axioms (see Section cooperative game theory definitions), and is the only payoff division that does so. The Shapley value is based on the idea that the payoff of the game should be divided such that each agent’s share is proportional to its contribution to the payoff. This characteristic is particularly useful in our problem, given that we would like to divide the discount based on how much each apartment has contributed to it. Therefore, we use the Shapley value to divide the total cost of cooling all apartments. As we will explain later, this then results in a fair division of the discount.

However, the use of Shapley value in our problem poses two computational challenges: (i) the time complexity of the Shapley value itself, which is exponential in the number of agents, and (ii) solving an exponential number of binary integer programs, each being an NP-hard problem, in order to find the value (i.e., consumption cost) of every possible coalition. To address the former issue, several algorithms have been proposed to approximate, rather than calculate, the Shapley value [20–24]. Most of these approximations work by considering only a sample of coalition values, thus avoiding the need to consider an exponential number of such values. However, to obtain a reasonable bound on the approximation, the sample would often consist of tens of thousands (or more) of coalition values. In our setting, even a single coalition value can be hard to compute. In such a case, sampling techniques will fail, and a fundamentally different approach is needed in order to tackle the computational challenge. As such, we focus on mitigating the second issue, by assuming that coalitions are *computationally bounded rational*. That is, a coalition would internalise its limited computational resources when making decisions. This is opposite to most typical scenarios where agents are assumed to be perfectly rational (i.e., they are able to maximise their utility), which may not be always possible due to the limited computational resources of agents.

In the demand response problem stated above, we trade off optimality for the feasibility of dividing the total cost in a fair way, we model coalitions as computationally bounded rational. That is, due to limited computational resources, the *rational value* of each coalition (i.e., the optimal solution to the coalition’s load optimisation problem) cannot be computed in a reasonable time. More specifically, building upon the bounded rationality model proposed by Sandholm and Lesser [25] we argue that, given a suboptimal value for each coalition, it is still possible to divide the discount in a fair way. Our proposition leverages the additivity axiom [19], whereby the Shapley value of a game can be represented as the sum of the Shapley values of some alternative games (see section shapley value of bounded rational agents). Based on this, we represent the Shapley value of the game where every coalition is assigned its rational value as the sum of the Shapley values of two games:

- The game where the value of any coalition is its *bounded rational value*—the total consumption of the coalition given the best suboptimal solution found to the load optimisation problem.
- The game where the value of any coalition is its *rationality discrepancy* (i.e., the difference between its rational and bounded rational values).

Importantly, the *bounded rational* coalition values are possible to calculate, while using the limited computational resources available the *rational* values and the *rationality discrepancy* are not. As such, we can only compute the Shapley value of the game with bounded rational coalition values. We call this the *bounded rational Shapley value*. We argue that this payoff division scheme is fair in that it produces a payoff distribution that results from the following procedure (which is impossible to carry out given the available limited resources):

- *Step 1*: Divide the rational value of the grand coalition (despite the fact that we do not know it) “fairly” among the agents, i.e., in a way that meets Shapley’s axioms. Intuitively, the share of each agent can be thought of as a *reward* for its contribution to the rational value.
- *Step 2*: Divide the rationality discrepancy of the grand coalition (which again is unknown) fairly among the agents, in a way that meets Shapley’s axioms. Intuitively, each share can be thought of as a *penalty* for contributing to the failure of finding the rational value in reasonable time. For instance, if the presence of an agent in any coalition consistently increases the rational discrepancy (e.g., due to the agent’s strict constraints which increase the time required to compute the rational value), then this agent will be penalised. Note that the penalty may be negative if coalition values represent a cost.
- *Step 3*: Assign to each agent its fair reward minus its fair penalty.

Given this division mechanism, we introduce two greedy algorithms for optimising the cooling plan of apartments individually and collectively. These algorithms will help us find a reasonably good (but not necessarily optimal) solution in a timely manner, and as we explain later, these algorithms have useful features that we exploit to significantly expedite the optimisation of coalition loads. In more detail, the first algorithm incrementally finds intervals in a given day in which switching on the air conditioner results in the largest reduction of the discrepancy between householder’s preferences and the estimated temperature during the comfort period. The second algorithm optimises the load of a coalition of apartments (subject to the predetermined threshold and individual temperature preferences) by shifting the load of apartments that are more flexible in terms of their preferences. This algorithm exploits the fact that the more flexible an apartment is, the easier its preferences are satisfied. Using these two algorithms, we can identify a suboptimal coordination of loads (leading to a potentially lower saving from the discount compared to the optimal solution), while satisfying the householders’ temperature preferences. Then, our bounded rationality proposition establishes that, it is possible to obtain a fair division of the discount using the Shapley value.

As we mentioned earlier, the bounded rational Shapley value requires a suboptimal value for *every* coalition. To obtain this value, we use the above greedy algorithms to determine the minimum cost of the coalition when they coordinate their loads. However, instead of running the algorithms over and over for every coalition, we show that this process can be carried out much more efficiently using a dedicated dynamic programming algorithm. This is because the information accrued from the optimised load of a coalition can be re-used in optimising the load of some other coalitions.

Finally, our experimental results evaluate how the costs incurred by an apartment vary, as its cooling preferences are changed, on a single day. We compare the costs that an apartment would be charged for its consumption in four different cases: (i) when the apartment does not sign up to the discount scheme and optimises its load independently, (ii) when the apartment optimises its load as a member of the coalition and and benefits from the discount, but only its consumption in the grand coalition is considered (unlike the Shapley value which considers all subsets of the grand coalition), (iii) when the apartment optimises its load as a member of the coalition and each apartment receives an equal share of the total saving from the discount (the difference between the payment of the grand coalition at the discounted and normal rates is equally divided and deducted from the payment of each apartment), and (iv) when the apartment optimises its load as a member of the coalition and receives its bounded rational Shapley value. The results show that with higher setpoint temperatures the payments drop, with higher rates of thermal leakage (poorer insulation) they increase, and with higher tolerances of setpoint deviation the payments decrease.

In summary, our main technical contribution in this paper includes: (i) exploiting an often less noted property of the Shapley value to extend it to games with bounded rationality, (ii) designing a demand response program to help mitigate peaks caused by cooling loads, (iii) designing two greedy algorithms to coordinate the loads of individual and groups of apartments to cap aggregate loads and avoid peaks, and (iv) developing a dynamic program for the greedy algorithms to speed up calculation of the Shapley value even further.

The rest of this article is organised as follows. Section cooperative game theory definitions introduces cooperative game theory definitions and provides a background on the Shapley value. Section coordinating cooling loads discount scheme formalises the discount scheme as a cooperative game. Sections thermal dynamics of apartments and user comfort model present formal models of thermal dynamics of apartments and the cooling preferences of users. In sections independent optimisation of loads and collective optimisation of loads we formalise the problem of optimising cooling loads of apartments individually and as coalitions. Section an example with three apartments provides an example of optimising the load of a block consisting of three apartments. In section computationally efficient optimisation of loads, we provide two greedy algorithms for efficient optimisation of loads. In section calculating payments using the Shapley value, we present the bounded rationality model and provide a dynamic programming algorithm to calculate the Shapley value efficiently. Section evaluation of the payments of apartments presents an experimental evaluation of our model and algorithms. Finally, section conclusions and future work concludes the article and states potential directions for future work.

## Cooperative game theory definitions

In this section, we introduce cooperative game theory notations and definitions, borrowed from [15], which we will refer to throughout the paper. Given a set of agents, *N* = {1, …, *n*}, a *coalition* *C* is a subset of *N*. The coalition *N* is referred to as the *grand coalition*. The value or worth of a coalition is expressed by a *characteristic function*, *v*, which maps each subset of *N* to a real number, i.e., $v:2^{N}\mapsto R$. A cooperative characteristic function game is specified using a pair, (*N*, *v*), consisting of the set of agents *N* and a characteristic function *v*. A *payoff distribution* is a vector *x* = [*x*_1_…*x*_*n*_], where *x*_*i*_ represents how much of the value of the coalition should be allocated to agent *i*. A game is *super-additive* if the value of every coalition is at least equal to the sum of the values of any two disjoint subsets of that coalition, i.e., ∀*C*, *D* ⊆ *N*;*C* ∩ *D* = ∅ ⇒ *v*(*C* ∪ *D*) ≥ *v*(*C*) + *v*(*D*).

### The Shapley value

In order for the agents to evaluate their prospects of playing a superadditive game, Shapley proposed a *value* [19], whereby agents receive a payoff equal to their value. More specifically, Shapley argued that a coalition of *n* agents can form in *n*! different ways (considering all the possible joining orders), and that in each order, as an agent steps in the coalition, it makes a *marginal contribution* to the agents who joined before it. The marginal contribution of agent *i* to coalition *C* is the difference in *C*’s value that is due to *i* joining *C*, i.e., it is equal to *v*(*C* ∪ {*i*}) − *v*(*C*). Shapley argued that the fair way to divide the payoff of the grand coalition is to allocate each agent its average marginal contribution in all joining orders. This solution concept is known as the Shapley value. More formally, the Shapley value of agent *i* in game (*N*, *v*) is calculated as:

$$\begin{array}{cc} \phi [i,v] & =\frac{1}{n!}\sum_{\pi \in \Pi (N)}[v\left(P_{i}^{\pi }\cup \{i\}\right)-v\left(P_{i}^{\pi }\right)] \end{array}$$ (1a)

$$\begin{array}{cc} & =\sum_{C\subseteq N\backslash \{i\}}\frac{|C|!\;(n-|C|-1)!}{n!}\left(v\left(C\cup \left\{i\right\}\right)-v\left(C\right)\right)\;, \end{array}$$ (1b)

where Π(*N*) is a set containing all possible permutation of agents, and $P_{i}^{\pi }$ is the set of agents that precede *i* in the permutation *π*. We will refer to the coefficient of the marginal contributions in the second formula using the notation *ω*. More formally, let *s* denote the size of coalition *C* in the marginal contribution *v*(*C* ∪ {*i*}) − *v*(*C*), this coefficient is given as:

$$\begin{array}{c} \omega \left(n,s\right)=\frac{s!\;(n-s-1)!}{n!}\; \end{array}$$ (2)

The Shapley value satisfies the axioms of *symmetry*, *efficiency*, *null-player*, and *additivity*. Symmetry states that if two agents make the same marginal contributions to any coalition, their values are equal. Efficiency implies that the value of the grand coalition is fully divided. The null-player axiom states that any player whose marginal contribution to every coalition is zero, would have a value of zero. Finally, the additivity axiom states that if a new game is obtained by adding the characteristic function of two different games with the same set of agents, the value of an agent in the new game is equal to the sum of its values in the two games. More formally:

- **Symmetry**: ∀*i*, *j* ∈ *N* ∀*C* ⊆ *N*\{*i*, *j*} *v*(*C* ∪ {*i*}) = *v*(*C* ∪ {*j*}) ⇔ *x*_*i*_ = *x*_*j*_;
- **Efficiency**: ∑_*i*∈*N*_ *x*_*i*_ = *v*(*N*);
- **Null player**: ∀*i* ∈ *N*∀*C* ⊆ *Nv*(*C* ∪ {*i*}) = *v*(*C*) ⇔ *x*_*i*_ = 0;
- **Additivity**: Let the game (*N*, *v*) be the *sum* of two other games, namely (*N*, *v*_1_) and (*N*, *v*_2_), i.e., ∀*C* ⊆ *N*, *v*(*C*) = *v*_1_(*C*) + *v*_2_(*C*). Furthermore, let *x*, *x*^1^, *x*^2^ denote the payoff distributions of (*N*, *v*), (*N*, *v*_1_), and (*N*, *v*_2_), respectively. Then, the following holds: $\forall i\in N,x_{i}=x_{i}^{1}+x_{i}^{2}$.

As is common in the literature, we say that a division of the grand coalition value is *fair* if it satisfies the above axioms. In fact, the Shapley value is the only value that satisfies them [19]. The Shapley value is also individual rationality in superadditive games (∀*i* ∈ *N*;*ϕ*[*i*, *v*] ≥ *v*({*i*})).

## Coordinating cooling loads discount scheme

Consider a set of *n* apartments in a block, indexed by *i* ∈ *N* ≔ {1, 2, …, *n*}. Denote by $l_{i}^{t}$ the cooling load of apartment *i* at time *t* (in kW), and denote by *p* the price at which every kWh is charged. In order to encourage consumers to use less energy for cooling, which constitutes a significant amount of the domestic load in warm-climate countries [6, 7], the electricity supplier offers a discount scheme. Specifically, each apartment in a block is offered a binary option of signing up to the scheme or not. Let *K* = {1, …, *k*} represent an entire day divided into *k* equal-length time slots, and $l_{i}=\left[l_{i}^{1}l_{i}^{2}\ldots l_{i}^{k}\right]$ represent the vector of cooling loads of apartment *i* in all time slots in *K*. If at any point in time throughout the day, the cooling load of the block is not more than *ψ* kW, i.e., $\forall t\in K;\sum_{i\in N}l_{i}^{t}\leq \psi$, then those apartment that have signed up are charged at *f* < *p* per kWh of usage, and the rest are charged at *p* per kWh. The cooling load of an apartment *i* at time *t* is:

$$\begin{array}{c} l_{i}^{t}=P_{i}\times \eta_{i}^{t}, \end{array}$$ (3)

where *P*_*i*_ is the electric power of the AC (in kW), and $\eta_{i}^{t}\in \left\{0,1\right\}$ represents a *cooling action*, which is a binary variable that indicates whether or not air conditioning has been used at time *t*.

Since the discount is offered only when the whole block’s load is below *ψ*, the price at which a coalition *C* ⊆ *N* is charged is also influenced by the behaviour of the apartments in the same block that have not subscribed to the scheme, i.e., *N*∖*C*. In cooperative game theory terms, the value of the coalition in this case is influenced by the agents outside it. If those apartments could form other coalitions, then we would have a game with *externalities* (also known as a partition function game), which is a game where the value of a coalition depends on how other agents are structured [26]. However, since the discount scheme does not cater for other arrangements, the apartments that do not sign up cannot form any other coalitions. Therefore, we have a special case of externalities where the agents outside the coalition can only be structured as singletons, and the game is reduced to a characteristic function game.

Naturally, each apartment, whether signed up to the scheme or not, would want to optimise its use of the AC such that its internal temperature preferences are satisfied with minimal electricity consumption. In order to secure the discount, those apartments that do sign up need to coordinate their loads so that the aggregate load will be kept below the threshold and their internal temperatures remain as they individually deem comfortable. Clearly, if an apartment decides not to sign up, it can only optimise its load independently, without any coordination with other apartments.

Assuming that some of the *n* apartments form a coalition, *C*, and sign up to the scheme, the aggregate cooling load of all *n* apartments (in kW) at time *t* is given by:

$$\begin{array}{c} l_{N}^{t}=l_{C}^{*t}+\sum_{i\in N\backslash C}l_{i}^{*t}, \end{array}$$

where $l_{C}^{*t}$ represents the aggregate optimal cooling load at time *t* of the apartments that have signed up, and $l_{i}^{*t}$ represents the optimal cooling of apartment *i*, at time *t*, which has not signed up.

Observe that a coalition can meet the threshold mostly through running its members’ ACs for longer periods (e.g., during off-peak times). Therefore, meeting the threshold potentially requires extra consumption of electricity, which would incur a higher cost. In theory, it is possible that the extra consumption becomes so high that even at the discounted rate the cost becomes higher than the sum of independent costs. This outcome is clearly not desirable, Therefore, if the discounted cost turns out to be higher, or if a feasible solution to the collective optimisation cannot be found, then the apartments optimise their loads independently. Based on this, we can write the optimal consumption of a coalition (measured in kWh) as:

$$\begin{array}{c} c\left(C\right)=\{\begin{array}{cc} \sum_{t\in K}l_{C}^{*t}\times \Delta t & \;\sum_{t\in K}l_{C}^{*t}\;\Delta t\;f\leq \sum_{i\in C}\sum_{t\in K}l_{i}^{*t}\;p\\ \sum_{i\in C}\sum_{t\in K}l_{i}^{*t}\times \Delta t & \;\sum_{t\in K}l_{C}^{*t}\;\Delta t\;f>\sum_{i\in C}\sum_{t\in K}l_{i}^{*t}\;p \end{array}, \end{array}$$ (4)

where Δ*t* is the duration of a time slot (in seconds). Based on the above consumption function, we now define the characteristic function, *v*, of the cooperative game (*N*, *v*) that represents the above discount scheme. In more detail, *v*(*C*) is equal to the total cost of consumption of its members. More formally, *v*(*c*) is given by:

$$\begin{array}{c} v\left(C\right)=c\left(C\right)\times \{\begin{array}{cc} f & \forall t\in K\;;\;l_{N}^{t}\leq \psi \\ p & \forall t\in K\;;\;l_{N}^{t}>\psi \end{array} \end{array}$$ (5)

With respect to the above characteristic function, it is clear that the apartments will not be worse off by joining the grand coalition. Therefore, it would be in the interest of all apartments to sign up and benefit from the potential discount.

In the next section, we explain how the cooling load of each apartment can be optimised so as to minimise the electricity consumption and satisfy its temperature preferences. We first present the model of thermal dynamics of an apartment that governs the evolution of its internal temperature.

## Thermal dynamics of apartments

We use a standard thermal model in which heat is assumed to enter an apartment (by thermal conduction) at a rate that is proportional to the temperature difference between the cold air inside and the hot air outside [27–29]. This model also incorporates the thermal capacity of the building structure, since through experimentation on real data collected from apartments in Jeddah, Saudi Arabia we found that this model best explains the observed data. This thermal model is represented as a set of coupled difference equations as per:

$$\begin{array}{cc} T_{int}^{t+1} & =T_{int}^{t}-r\eta^{t}\Delta t+\alpha \Delta t(T_{env}^{t}-T_{int}^{t})\\ T_{env}^{t+1} & =T_{env}^{t}+\beta \Delta t(T_{int}^{t}-T_{env}^{t})+\gamma \Delta t(T_{ext}^{t}-T_{env}^{t}), \end{array}$$ (6)

where $T_{int}^{t}\in R^{+}$ denotes the internal temperature (measured in °C) of apartment *i* at time *t*, $T_{env}^{t}\in R^{+}$ denotes the temperature of the building structure, or envelope, (measured in °C), and $T_{ext}^{t}\in R^{+}$ denotes external temperature (measured in °C). We assume that $T_{ext}^{t}$ is the same for all apartments in *N*. Furthermore, *r* (measured in °C/hr) represents the rate at which the AC reduces the internal temperature, and *α*, *β* and *γ* (measured in 1/hr) are the rates of leakage from the envelope to the inside, from the inside to the envelope, and from the outside to the envelope, respectively. Hereafter, when we refer to an apartment in a coalition we index the notation by *i*.

Eq (6) is the discrete equivalent to a set of coupled differential equations which has been used previously to model data collected from real buildings [30]. In this model, an envelope is introduced to act as an additional thermal mass to minimise internal temperature changes due to extremes of temperature outside. Given historical observations of $T_{int}^{t}$ and $T_{ext}^{t}$, and the times during which the AC was on (which we collected from a number of apartments in Jeddah, Saudi Arabia) we predicted the evolution of the internal temperature, ${\bar{T}}_{int}^{t}$. The error in this prediction is given by $\sum_{t\in K}{\left({\bar{T}}_{int}^{t}-T_{int}^{t}\right)}^{2}$. Consequently, the best estimates of the parameters are those that minimise this error and can be learned through recursive least squares [31].

## User comfort model

We outline a few assumptions that underpin the operation of the cooling system in an apartment. We assume that an apartment has a central air conditioning driven by a heat pump that transfers heat from a lower temperature heat source (the apartment) into a higher temperature heat sink (external ambient air). This system is connected to a thermostat within the apartment, where a user can set a desired temperature to be maintained, i.e., the setpoint temperature, denoted as *T*_*set*_ (°C).

The user in each apartment can specify the time interval during which “comfort” is desired. That is, the time slots when the user wants the internal temperature to be maintained at, or close to, *T*_*set*_. We refer to this interval as the *comfort period*, and define it as: *H* = {*t* ∈ *K*|*CST* ≤ *t* ≤ *CET*}, where *CST* ∈ *K* and *CET* ∈ *K* are the comfort start time and comfort end time, respectively. A tolerance level is specified by the user to limit deviations of the internal temperature from the setpoint temperature, during the comfort period. We denote this tolerance by $\theta \in R^{+}$ (°C). Note that lower values of *T*_*set*_ suggest that a user feels more comfortable at lower temperatures, and smaller values of *θ* indicate that a user is sensitive to deviations of the temperature from the setpoint.

**Fig 1:**
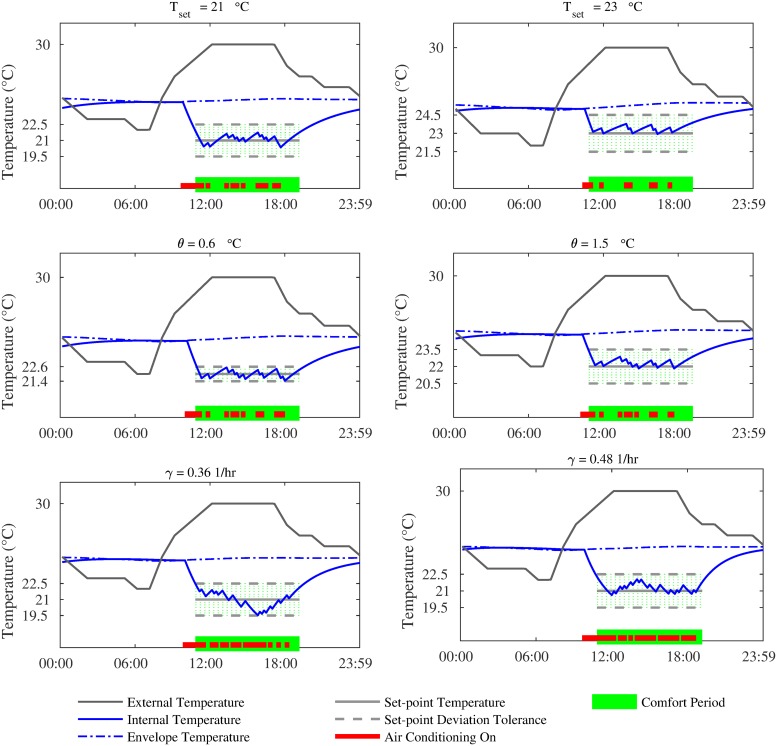
The impact of varying *T*_*set*_, *θ* and *γ* on the temperature and cooling profiles.

Intrinsically, the above preferences have an impact on the cooling load (Eq (3)). Fig 1 shows the effect of varying *T*_*set*_, *θ* and *γ* on the temperature and cooling profiles. The bottom of each subplot shows the cooling actions over the course of a day. As *T*_*set*_ is lowered, the amount of cooling required increases proportionately to achieve lower temperatures. As can be seen in the corresponding plots in Fig 1, more cooling is required when *T*_*set*_ = 21°C than internal temperature profile in an apartment when *T*_*set*_ equals 21°C *T*_*set*_ = 23°C. The total time when the AC is on is 52% less in the latter case. Similarly, when *θ* is small, a user is more sensitive to deviations of the internal temperature from *T*_*set*_. Consequently, the AC is turned on for longer to ensure that the deviation of the internal temperature from *T*_*set*_ lies within the tolerance level, resulting in higher energy consumption. This is evident in Fig 1, where the *θ* is set to 0.6°C and 1.5°C. As can be seen, more cooling is required for a larger *θ*. The total time when the AC is on is 12% less in the former case. Furthermore, as per Eq (6), an apartment that is well-insulated will have a small *γ* value, whereas a leaky apartment will have a high value. This observation is of interest as more cooling is required to maintain a leaky apartment at a certain temperature. This effect is evident in Fig 1, where the value of *γ* used to generate the plots are 0.36 1/hr and 0.48 1/hr, respectively. The total amount of time when the AC is on is 24% more for *γ* = 0.48.

## Independent optimisation of loads

Having introduced the comfort model, we describe how an apartment optimises its use of the AC so as to satisfy only its own comfort preferences. In summary, as we described in section user comfort model, the preferences of an apartment are: (i) the desired setpoint temperature, denoted by *T*_*set*_, (ii) a tolerance level on the deviation of the internal temperature from the setpoint, denoted by $\theta \in R^{+}$, and (iii) the comfort start time (*CST*) and comfort end time (*CET*) which determine *H*—the set of time slots representing the comfort period. Based on these, we define an optimal cooling plan for apartment *i* to be a vector of cooling actions [*η*^1^ *η*^2^…*η*^*k*^], that result in meeting the above preferences as well as the following requirements: (i) the overall energy consumption is minimised, i.e., all constraints are satisfied with the AC running in as few time slots as possible, (ii) the internal and envelope temperatures at the start and end of the day converge. Note that the latter requirement is to ensure that the cooling plan is optimised over an infinite horizon, which prevents erroneous solutions that minimise AC use in the short term, but require additional cooling later, as would be the case if a finite planning horizon were used. Given the above preferences and requirements, the optimal cooling load of an apartment throughout the day can be computed as per Eq (3) using *η*^1^, *η*^2^, …, *η*^*k*^ found by solving the following optimisation problem:

$$\begin{array}{c} \begin{array}{ccc} & \text{minimise}\sum_{t\in K}\eta^{t}\\ & \text{subject}\;\text{to}\;\forall h\in H,\;\;|T_{int}^{h}-T_{set}|\leq \theta , & \text{and}\\ & T_{int}^{1}=T_{int}^{k}\;,\;T_{env}^{1}=T_{env}^{k}. \end{array} \end{array}$$ (7)

Observe that our formulation avoids the explicit trade-off between consumption and comfort within a single objective function, which is dependent on specifying appropriate weights for both objectives. This is because, in practice, there is no principled way to specify such weights [32].

## Collective optimisation of loads

We now describe how a coalition of apartments, *C*, collectively optimise their cooling loads. Similar to the single apartment case, the user in each apartment in *C* specifies their individual cooling preferences. These include their desired setpoint temperature, *T*_*set*_[*i*], their tolerance on the deviation of the internal temperature during the comfort period, *θ*_*i*_, and their comfort start and end times which determine *H*_*i*_. In finding an optimal cooling plan for the coalition, we introduce a key additional constraint, which ensures that at all times, the total load of all apartments in the coalition, plus the total load of the apartments who optimise their loads independently, is less than or equal to *ψ*. More formally, the vector of optimal cooling actions, $\left[\eta_{i}^{1}\eta_{i}^{2}\ldots \eta_{i}^{k}\right]$, for every apartment *i* ∈ *C* is given by the following optimisation problem:

$$\begin{array}{c} \begin{array}{ccc} \forall i\in C\;\; & \text{minimise}\sum_{t\in K}\eta_{i}^{t}\;\text{subject}\;\text{to:}\\ & \forall h\in H\;\;|T_{int}^{h}\left[i\right]-T_{set}\left[i\right]|\leq \theta_{i}, & \text{and}\\ & \forall t\in K\;\;l_{N}^{t}=\sum_{i\in C}P_{i}\;\eta_{i}^{t}+\sum_{j\in N\backslash C}l_{j}^{*t}\leq \psi , & \text{and}\\ & T_{int}^{1}\left[i\right]=T_{int}^{k}\left[i\right]\;,\;T_{env}^{1}\left[i\right]=T_{env}^{k}\left[i\right]. \end{array} \end{array}$$ (8)

If a feasible solution to the above optimisation did not exist, the apartments would optimise their loads individually as per Eq (7). Note that it is possible for an individual apartment in *C* to have a significant impact on the feasibility of *C* satisfying the threshold constraint. For instance, if *T*_*set*_[*i*] is set to a particularly low temperature, or *θ*_*i*_ is particularly small, the corresponding energy consumption in that apartment will be greater, which in turn increases the likelihood of the aggregate load exceeding the threshold. As the individual apartments become more flexible and less stringent with their preferences, the aggregate cooling load, $l_{N}^{t}$, is more likely to satisfy the constraint on the threshold.

## An example with three apartments

Having established the theoretical underpinnings of how cooling loads are independently and collectively optimised, we now illustrate how they work in practice through a simple example. Consider a 3-player game (*N* = {1, 2, 3}), where a block consists of three apartments. All three apartments agree to participate in the discount scheme. Apartment 1 desires that the temperature be maintained at 21°C (*T*_*set*_[1] = 21°C) for 6 hours from *CST* = 10:00 to *CET* = 16:00, and is satisfied with wide swings of temperature (*θ*_1_ = 1.5°C). Apartment 2 desires the temperature to be at 22°C (*T*_*set*_[1] = 22°C) for 8 hours from *CST* = 09:00 to *CET* = 17:00, and has very strict preferences over temperature (*θ*_2_ = 0.5°C). Apartment 3 too desires the temperature to be at 21°C (*T*_*set*_ [3] = 21°C) from *CST* = 10:00 to *CET* = 16:00, and is satisfied with wide swings of temperature (*θ*_1_ = 1.5°C).

We assume that the AC in each apartment operates on a 10-minute cycle, i.e., a cooling decision is made for each 10-minute interval in a day (Δ*t* = 600s). As a result, *K* = [1, …, 144] and the decision variable is $\eta_{i}^{t}$, ∀*t* ∈ *K*. We also assume that the ACs in all three apartments are similar and consume at a rate of 3 kW when on, i.e. *P*_*i*_ = 3 kW. Hence, the total possible energy load of all three apartments if they optimise their loads independently is 9 kW. Now, when the apartments participate in the scheme, a threshold at 3 kW (i.e., a $\frac{2}{3}$ reduction), is set on their total load.

**Fig 2:**
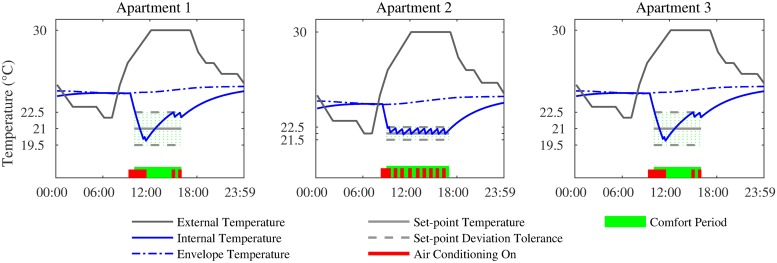
The temperature and cooling profiles when loads are optimised individually.

**Fig 3:**
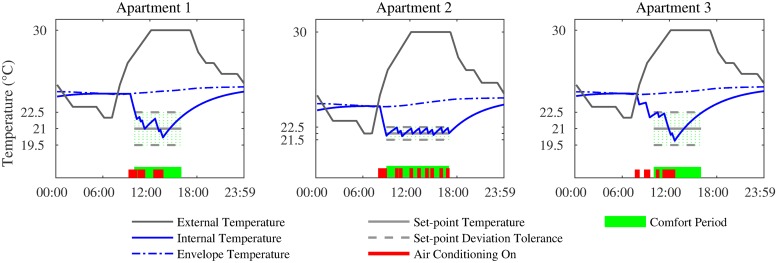
The temperature and cooling profiles when loads are optimised collectively.

We first consider the case where all 3 apartments optimise their cooling loads independently. The bottom of plots in Fig 2 show the individual cooling profiles of each apartment for a single day. Each profile is obtained by solving the optimisation problem in Eq (7) using CPLEX, to yield $\eta_{i}^{t}$ (∀*t* ∈ *K*). Also shown in Fig 3 are the corresponding internal temperature profiles, which are estimated by iterating Eq (6) for each apartment, using the cooling actions, $\eta_{i}^{t}$ as inputs. It is evident from the plot that the internal temperature is within the desired setpoint deviation tolerance at times when the users desire cooling. Also, this deviation is greater in Apartment 1 and 2, as they are less sensitive to large swings in temperature. Finally, the optimisation ensures that the temperature at the start and end of each day is the same, as required.

Next, we consider the case where all 3 apartments form a coalition and collectively optimise their loads to ensure that their aggregate load does not exceed the threshold. The bottom of plots in Fig 3 show the cooling profiles of each apartment for a single day. Each profile is obtained by solving the optimisation problem in Eq (8) using CPLEX, to yield $\eta_{i}^{t}$ (∀*t* ∈ *K*), which in turn generate a temperature profile based on the thermal model as per Eq (6).

**Fig 4:**
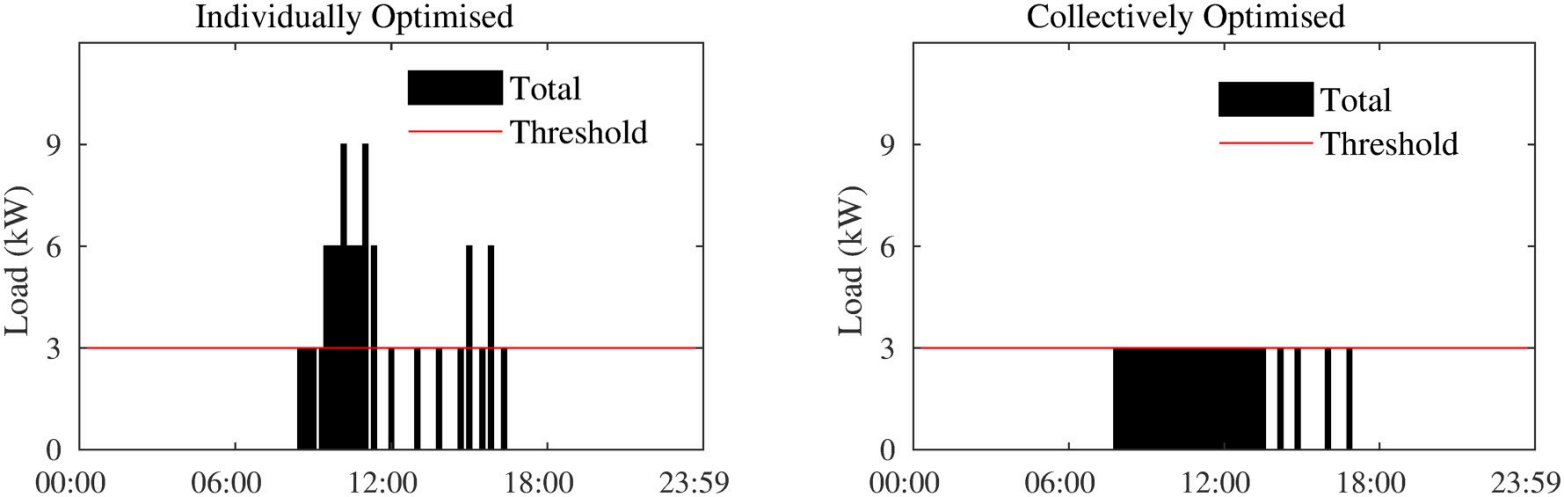
Total load when loads are optimised individually and collectively.

More importantly, as shown in Fig 4, the collective optimisation results in the aggregate never exceeding the threshold during the day, making the block eligible for the discount. This is not the case when optimised independently.

Since optimising the loads of the three apartments is tractable, we can readily divide the total cost of the apartments based on the Shapley value as per Eq (1b). We assume that the electricity cost is $0.15 per kWh when apartments optimise their cooling loads independently. As per the discount scheme, if they ensure that their aggregate load does not exceed 3 kW, then the electricity cost will be reduced to $0.08 per kWh.

When optimised collectively to keep the aggregate load below the threshold, Apartment 2’s preferences are so strict that a somewhat small change is only possible. However, Apartment 1 and Apartment 3 are able to shift their cooling loads to satisfy their preferences as well as the threshold constraint, but to do so, they have to run their AC earlier and longer. These are naturally reflected in the value of each coalition as shown in Table 1. As can be seen, all apartments would incur the same cost if they did not participate in the scheme. However, when they do participate, interestingly, Apartment 2’s share of the discount becomes slightly less than that of the other two. This is due to its stricter preferences that are harder to satisfy in the collective optimisation. Also note that the optimal loads of Apartment 1 and Apartment 3, who have identical preferences, are the same. Therefore, all of their marginal contributions (and their Shapley values, in turn) are equal. Based on the coalition values given in the table and the Shapley value formula (Eq 1b), the payments of apartments 1, 2 and 3, are $3.08, $3.20 and $3.08, respectively.

**Table 1:** The cost of coalitions when members optimise their loads collectively and independently.

Coalition (*C*)	Threshold	Cost	Independent Apartments (*N*∖*C*)	Cost
{}	Not Satisfied	$0	{1},{2},{3}	$17.55
{1}	Not Satisfied	$5.85	{2}, {3}	$11.70
{2}	Not Satisfied	$5.85	{1}, {3}	$11.70
{3}	Not Satisfied	$5.85	{1}, {2}	$11.70
{1, 2}	Satisfied	$6.24	{3}	$5.85
{1, 3}	Satisfied	$6.00	{2}	$5.85
{2, 3}	Satisfied	$6.24	{1}	$5.85
{1, 2, 3}	Satisfied	$9.36	{}	$0

## Computationally efficient optimisation of loads

In the simple example of three apartments, optimising the cooling loads did not require a considerable amount of computation, and calculating the Shapley value was easy. However, as more apartments are added to the game, satisfying the constraints takes considerably more time. Considering the fact that, in calculating the Shapley value, an exponential number of coalitions must be optimised, the time it takes to calculate the payoff of only a few agents can be very long. In order to overcome this, we next present two greedy algorithms, namely, iOPT and cOPT, for optimising the cooling load of the apartments independently and collectively. Due to the difficulty of finding the optimal solutions in reasonable time, we use these algorithms and trade off optimality for computation speed. Thus, we consider a feasible cooling plan given by these algorithms to be the best solution we can find given our limited computational resources, which may or may not coincide with the actual optimal solution. Given this, we call such a solution *best-found*.

**Algorithm 1** Greedy Algorithm For Optimising Apartments Independently

**function** iOPT (*T*_*set*_, *θ*, *T*_*ext*_, Λ, *maxIterations*)

*η* ← []

**for** *iteration* = 1 to *maxIterations* **do**

 *bestTime* ← −1

 *minDiscomfort* ← ∞

 *maxDeviation* ← 0

 **for** *t* = 0 to *k* **do**

  **if** *t* ∈ Λ **then**

   **continue**

  **end if**

  *η*_*test*_ ← *η*

$\eta_{test}^{t}\leftarrow 1$

  ∀*t* ∈ *K* update $T_{int}^{t}\left[test\right]$ and $T_{env}^{t}\left[test\right]$ as per Eq (6)

  Calculate Δ*D*_*test*_

  **if** Δ*D*_*test*_ < *minDiscomfort* **then**

   *bestTime* ← *t*

   *minDiscomfort* ← Δ*D*_*test*_

  **end if**

 **end for**

 **if** *bestTime* > −1 **then**

  *η*^*bestTime*^ ← 1

  ∀*t* ∈ *K* update $T_{int}^{t}$ and $T_{env}^{t}$

 **end if**

**end for**

**return** *maxDeviation* ≤ *θ*

First, we explain the workings of iOPT, the pseudocode of which is presented in Algorithm 1. Using a heuristic, iOPT searches for a set of cooling actions that satisfy the constraints of an individual apartment as per Eq (7). This heuristic, which we call the *discomfort* of an apartment, represents the discrepancy between an apartment’s preferences (as outlined in section user comfort model) and the temperature profile resulting from a cooling plan found by the algorithm. More formally, the discomfort of apartment *i*, denoted by Δ*D*_*i*_, is the largest deviation of the internal temperature from the setpoint temperature during the comfort period. This is given as:

$$\begin{array}{c} \Delta D_{i}=\max_{h\in H}\;\left(T_{int}^{h}\left[i\right]-T_{set}\left[i\right]\right) \end{array}$$ (9)

The algorithm incrementally finds the time slots where switching the AC on results in the largest discomfort reduction. Initially, the AC is off in all time slots (i.e., $\forall t\in K,\eta_{i}^{t}=0$), and is then switched on only if it results in a reduction of the discomfort. This way, in addition to searching for a feasible solution, the consumption is also minimised (as required in the optimisation problem in Eq (7)). However, as soon as the constraints of the apartment are satisfied, the algorithm will not seek to minimise the consumption further. Since this procedure is indepedent of the number of apartments, the time complexity of the algorithm is constant. To be more exact, it is a function of |*K*|^2^, which implies even with finer time resolutions the computation will increase very reasonably. Similarly, the space complexity of iOPT is independent of *n*, and is only a multiple of |*K*|, which is also constant.

Furthermore, recall that one of the constraints in Eq (7) is that the internal and envelope temperatures at the start and end of the day should converge (i.e., $T_{int}^{1}=T_{int}^{k}$ and $T_{env}^{1}=T_{env}^{k}$). To ensure this, one can run Algorithm 1 repeatedly, and in each iteration calculate $T_{int}^{1}$ based on $T_{int}^{k}$ from the previous iteration. We have found through experiments that, this way, no more than 4 iterations are typically needed for the internal and envelope temperatures at the end of the day to be within 0.1°C of the start of the day. Moreover, if iOPT does not find a feasible solution, the algorithm will terminate after *maxIterations* iterations. Lastly, iOPT takes a set of time slots Λ as input, which, as we explain later, is used in cOPT to indicate the time slots in which the aggregate load is greater or equal to the threshold. When an apartment is optimised independently, this set is empty.

If iOPT finds a feasible solution for apartment *i*, it gives a vector of $\eta_{i}^{t}$ values, based on which we calculate the best-found cooling load of the apartment when is independent of the coalition. This load is then used to calculate the consumption of apartment *i* as per Eq (4), based on which we obtain the bounded rational value of the singleton {*i*}, i.e., *v*^*BR*^({*i*}). Next, we describe the workings of cOPT, which optimises the cooling load of the members of a coalition. The pseudocode is given in Algorithm 2.

**Algorithm 2** Algorithm For Optimising Apartments Collectively

**function** cOPT (*C*, *N*, *ψ*, *T*_*set*_, *θ*, *T*_*ext*_, *maxIterations*)

**for all** *i* ∈ *N* **do**

 iOPT(*T*_*set*_[*i*], *θ*_*i*_, *T*_*ext*_, ∅, *maxIterations*)

**end for**

**if** $\forall t\in K\;l_{N}^{t}\leq \psi$ **then**

 **return** true

**end if**

// *C* is sorted beforehand based on Eq (10)

**for all** *i* ∈ *C* **do**

$\Lambda \leftarrow \{t\in K|\;l_{N}^{t}\geq \psi \}$

 *successfullyReOptimised*← iOPT (*T*_*set*_[*i*], *θ*_*i*_, *T*_*ext*_, Λ, *maxIterations*)

 **if** *successfullyReOptimised* **then**

  **if** $\forall t\in K\;l_{N}^{t}\leq \psi$ **then**

   **return** *true*

  **end if**

 **else**

  Revert *i* to its individually optimised state

 **end if**

**end for**

**return** *false*

Given a coalition, all member apartments are first independently optimised using iOPT. If by doing so the constraints of all apartments, as well as the threshold constraint are already satisfied, then the best-found cooling plan of the members of the coalition, in this case, is the same as when the apartments optimise their loads independently. However, if the threshold is not satisfied, it means that at least in one time slot there is congestion, i.e., the aggregate load is higher than the threshold. We denote the set of *congested time slots* by Λ, which is formally defined as: $\left\{t\in K|l_{N}^{t}\geq \psi \right\}$. The objective of the algorithm is to *decongest* these time slots by *re-optimising* at least some of the apartments such that they do not run their ACs in these time slots. Re-optimising an apartment given a set of congested time slots means that after all apartments are initially independently optimised, the apartment in question is again optimised using iOPT, such that its AC is not turned on in any of the congested time slots. Obviously, those members of the coalition that are stringent with their temperature preferences may not be able to avoid the congested time slots. As such, the algorithm performs decongestion with respect to the flexibility of the load of the apartments. The idea is that the more flexible an apartment is, the more likely it can satisfy its constraints without having to run its AC in the congested time slots. Observe that the longer the comfort period is, the more cooling is needed. Furthermore, as we saw in Fig 1, the higher the tolerance on the setpoint temperature of an apartment is, the less cooling it requires, and thus, it can be considered more flexible than an apartment that has a lower tolerance. Based on these observations, we use the following ratio as a heuristic to determine the severity of the preferences of the apartments relative to one another:

$$\begin{array}{c} \frac{\theta_{i}}{P_{i}\times \left|H_{i}\right|} \end{array}$$ (10)

Using the above heuristic, we initially sort the apartments in the coalition in ascending order, so that the least flexible apartment is dealt with first. Given a set of congested time slots, cOPT iteratively re-optimises the apartments using iOPT. In each iteration, the set of congested time slots, Λ, is computed anew. If Λ is not empty (i.e, the threshold constraint has not been satisfied yet), iOPT will be called again to optimise the apartment in the current iteration without being allowed to run its AC in the congested time slots. If the constraints of the apartment are successfully satisfied this way, the algorithm moves on to the next member, and repeats this procedure until the threshold is satisfied or all apartments have been re-optimised. In any iteration, if the constraints of the apartment are not successfully satisfied, its best-found cooling plan (as a member of the coalition) will be reverted to its independently optimised case. Similarly, if at the end of the process the threshold constraint is not satisfied, the best-found cooling plan of all apartments will be reverted to their independently optimised cases. Since cOPT deals with each member of *C* at most once, its time complexity is a linear function of |*C*|. With the average coalition size being (*n* + 1)/2, the time complexity complexity is *O*((*n* + 1)/2) in the average case. As for memory requirement, for each member of *C*, we do not need to store more than what iOPT requires. Therefore, the space complexity is again a linear function of |*C*|, which in the average case is *O*((*n* + 1)/2).

The effect of this collective coordination on individual apartments is that those that are more flexible turn out to lower their internal temperature ahead of their comfort period, so that they can avoid running their AC in the congested time slots. This process can potentially result in an incremental reduction of the congestions, until the threshold is eventually satisfied. While the end result may not be optimal, this ensures that joining the grand coalition will not make any member worse off in any case, making it rational for them to sign up to the discount scheme.

## Calculating payments using the Shapley value

Recall from section coordinating cooling loads discount scheme that the value of a coalition is given by the sum of the consumption of its members, when they collectively optimise their loads. Furthermore, we explained how a coalition can optimise its cooling plan in a computationally efficient manner. Therefore, based on the best-found cooling plans, we can calculate the coalition values and in turn the Shapley value of apartments, which is what they must pay for their consumption. In the next subsection, we argue why using the Shapley value based on the best-found cooling plans still results in a fair division of the discounted total cost. We then explain how the Shapley value in this setting can be efficiently calculated.

### Shapley value of bounded rational agents

A common assumption in cooperative game theory literature is that the characteristic function has a negligible computational cost, e.g., it can be done in constant time. However, in many real world problems, such as our discount scheme, and those considered in [33, 34], computing the value of a coalition *C* involves solving a hard optimisation problem. In our setting, *v*(*C*) is hard to compute optimally. As such, we will refer to *v*(*C*) as the *rational value* (rather than simply the *value*) of *C*. The corresponding *bounded rational game* of (*N*, *v*) is a new game, denoted by (*N*, *v*^*BR*^), where *v*^*BR*^(*C*) is the best-found solution to the cooling plan of *C* that is obtained given the available computational resources. We will refer to *v*^*BR*^(*C*) as the *bounded rational value* of coalition *C*.

Under the full rationality assumption, the rational value of every coalition is known, and thus, the value of the game can be fairly divided using the Shapley value. However, with bounded rational agents, the rational values of the coalitions are unknown, and it is not immediately clear how a fair division of the value of the game can be obtained. Next, we propose one way to deal with this issue.

We assume that it is possible to find a suboptimal solution to the cooling plan of every coalition in reasonable time (i.e., the bounded rational value). We also assume that an equal amount of computational resource is dedicated to calculating the value of each coalition. Note that this assumption does not imply that the cost of finding the bounded rational value of all coalitions is the same. Furthermore, in order to treat all agents without discrimination, we assume that the algorithm by which the coalition values are found is the same for all coalitions.

**Proposition 1**. *Given a game*, (*N, v*), *by allocating to each agent its Shapley value of the corresponding bounded rational game*, (*N*, *v*^*BR*^), *a payoff division is obtained that is fair in the following sense: (i) it fairly allocates each agent a payoff for its contribution to the total value of the game, i.e*., *v*(*N*), *(ii) each agent is fairly penalised for contributing to v*(*N*) *not being equal to v*^*BR*^(*N*).

Let us introduce some additional notation to better understand the intuition behind this proposition. For every *C* ⊆ *N*, let *v*^*RD*^(*C*) denote the *rationality discrepancy* of coalition *C*, defined as the difference between the rational and bounded rational values of *C*. This can be viewed as a penalty that *C* as a whole has to pay due to its members’ lack of full rationality. More formally,

$$\begin{array}{c} v^{RD}\left(C\right)=\left|v\left(C\right)-v^{BR}\left(C\right)\right|. \end{array}$$ (11)

From Eq (11) it is evident that the game (*N*, *v*) can be written as the sum of (*N*, *v*^*BR*^) and (*N*, *v*^*RD*^). Therefore, based on the additivity axiom of the Shapley value, the following holds:

$$\begin{array}{c} \phi \left[i,v^{BR}\right]=\phi \left[i,v\right]\pm \phi \left[i,v^{RD}\right]. \end{array}$$ (12)

The sign in the above equation is negative or positive depending on whether *v* represents profit or cost, respectively. While both *ϕ*[*i*, *v*] and *ϕ*[*i*, *v*^*RD*^] are unknown by assumption, it is possible to calculate *ϕ*[*i*, *v*^*BR*^]. Importantly, this is in fact agent *i*’s fair share of the total cost of the grand coalition (i.e., *v*(*N*)), which also factors in agent *i*’s fair share of the penalty due to its bounded rationality.

The implication of Proposition 1 is that when agents cannot find an optimal solution, they can still expect to receive a fair share of *v*^*BR*^(*N*). This is because *ϕ*[*i*, *v*^*BR*^] also satisfies the Shapley value axioms, and is the only value that does so.

Intuitively, the more rational a coalition’s members are (i.e., more computational resources are used to optimise their cooling loads), the smaller the rationality discrepancy of the coalition is. Therefore, if the agents in a game were fully rational, they would be able to diminish their penalty completely (i.e., *v*^*RD*^(*C*) would be zero), and thus, *ϕ*[*i*, *v*^*BR*^] would become exactly equal to *ϕ*[*i*, *v*] for all agents.

To illustrate Proposition 1 further, consider an example of a block consisting of three apartments that participate in the discount scheme. Through optimal coordination of their cooling loads, they are able to cap their aggregate load and achieve a discount of 50% per unit of electricity consumed. However, suppose that due to the thermal characteristics of the apartments and their temperature preferences, finding an optimal coordination in practice is so complex that they can manage to meet the threshold requirement, but can only approximately minimise their consumptions. As a result, the cost associated with coalitions is potentially higher than what would be possible if coordinating their loads were easier or more computational resources were available.

Now, to highlight the significance of Proposition 1, let us assume that Apartment 1 and 2 are identical in every respect, except that Apartment 1 is less flexible with its temperature preferences. Consequently, if computational resources were unlimited and its load were optimally coordinated with others, its cooling plan and temperature profile would be identical to those of Apartment 2. However, since in practice computational resources are limited, Apartment 1’s stricter preferences require more computation to satisfy compared to Apartment 2. A set of example values that describe this scenario, and the corresponding Shapley values are shown in Table 2.

**Table 2:** Payments according to Proposition 1 in an example scenario.

*C*	Threshold	*v*(*C*)	*v*^*BR*^(*C*)	*v*^*RD*^(*C*)
{}	Not Satisfied	$0	$0	$0
{1}	Not Satisfied	$10	$10	$0
{2}	Not Satisfied	$10	$10	$0
{3}	Not Satisfied	$18	$18	$0
{1, 2}	Not Satisfied	$20	$20	$0
{1, 3}	Satisfied	$15	$18	$3
{2, 3}	Satisfied	$15	$16	$1
{1, 2, 3}	Satisfied	$24	$28	$4

*i*	*ϕ*[*i*, *v*]	*ϕ*[*i*, *v*^*BR*^]	*ϕ*[*i*, *v*^*RD*^]
1	$7.50	$9	$1.50
2	$7.50	$8	$0.50
3	$9.00	$11	$2.00

It is immediately clear that for all three apartments, Eq (12) holds. Note that when agents are rational, Apartment 1 and 2 have identical values and marginal contributions, and therefore equal Shapley values. However, when they are bounded rational, the higher complexity of coordinating Apartment 1’s load with others means that Apartment 2 deserves a lower cost than Apartment 1, which is reflected by their bounded rational Shapley values. Proposition 1 establishes the fairness of this division. Apartment 1 and 2 are both fairly allocated a cost for contributing to the cost of any coalition in which they are involved. Simultaneously, they are individually penalised proportional to their contribution to the discrepancy between *v*^*BR*^ and *v* in any coalition in which they are involved. Moreover, Apartment 3, which has a higher individual cost than the other two in the rational case, still receives a portion of the discount when is bounded rational because it is able to coordinate its load when it is in a coalition with others.

### Efficient implementation of the Shapley value

Calculating the bounded rational Shapley value of all agents using the standard formula, i.e., Eq (1b), requires computing the value of each coalition multiple times. This is because in the standard formula, for each agent, one must iterate through all *C* ⊆ *N*, and in doing so *v*(*C*) is used multiple times. Therefore, the time complexity of computing the Shapley value for all agents using the standard implementation is *O*(*n* × 2^*n*^ × *O*(*v*)). This is not an issue in games where the characteristic function does not have a considerable computational complexity. However, given the bounded rationality assumption, computing *v*(*C*) more than once is highly costly. One trivial way to overcome this is to store all coalition values in memory, but doing so requires exponential memory space. Alternatively, instead of iterating through agents, we can iterate through all subsets of *N*, and in each iteration, we calculate the value of the subset. Then, we use this to update *all* marginal contributions of *all* agents that require that value. This ensures that the value of any given coalition is computed exactly once. This results in a time complexity of *O*(2^*n*^ × *O*(*v*)), at no additional memory space cost. Next, we explain this idea in more detail.

First, observe that calculating the marginal contribution of agent *i* to a coalition *D* requires the following two terms: *v*(*D* ∪ {*i*}) and *v*(*D*), which represent the value of the coalition *with* and *without* the agent, respectively. More formally:

$$\begin{array}{c} MC_{i\to D}=v\left(D\cup \left\{i\right\}\right)-v\left(D\right). \end{array}$$ (13)

For each *C* ⊆ *N*, we can use *v*(*C*) to calculate the “with agent” and “without agent” terms in marginal contributions of two groups of agents:

- ∀*i* ∈ *C* since ∃*D* ⊂ *C* s.t. *D* ∪ {*i*} = *C*; *v*(*C*) corresponds to the “with agent” term.
- ∀*i* ∈ *N*∖*C*; *v*(*C*) corresponds to the “without agent” term.

Furthermore, recall that in Eq (1b), each marginal contribution is multiplied by a weight given by Eq (2). If we multiply each of the two terms above by its corresponding weight, they become *ω*(*n*, |*D*|)*v*(*D* ∪ {*i*}) and −*ω*(*n*, |*D*|)*v*(*D*). Therefore, to calculate the Shapley value, we sum all coalition values multiplied by their corresponding weight just as in Eq (1b):

- For every non-empty coalition *C* ⊂ *N*, *v*(*C*) is multiplied by *ω*(*n*, |*C*| − 1) for every *i* ∈ *N*∖*C*, and multiplied by −*ω*(*n*, |*C*|) for every *i* ∈ *C*, *v*(*C*).
- For *C* = ∅, *v*(*C*), which only represents the value of a coalition without the agent, is multiplied by −*ω*(*n*, 0).
- For *C* = *N*, *v*(*C*), which only represents the value of a coalition with the agent, is multiplied by *ω*(*n*, *n* − 1).

The pseudocode of this process is presented in Algorithm 3.

**Algorithm 3** Efficient Implementation of the Shapley Value

**function** ShapleyValue(*N*, *v*)

*ϕ* ← [];

∀*i* ∈ *N*, *ϕ*[*i*, *v*] ← 0;

**for all** *C* ⊆ *N* **do**

 *weightWithAgent* ← *ω*(|*N*|, *max*(|*C*| − 1, 0));

 *weightWithoutAgent* ← −*ω*(|*N*|, *min*(|*C*|, |*N*| − 1));

 **for all** *i* ∈ *N* **do**

  **if** *i* ∈ *C* **then**

   *ϕ*[*i*, *v*] ← *ϕ*[*i*, *v*] + (*weightWithAgent* × *v*(*C*));

  **else**

   *ϕ*[*i*, *v*] ← *ϕ*[*i*, *v*] + (*weightWithoutAgent* × *v*(*C*));

  **end if**

 **end for**

**end for**

**return** *ϕ*

### Efficient calculation of the Shapley value using dynamic programing

In calculating the bounded rational Shapley value, when cOPT is sequentially applied to the subsets of the grand coalition, some steps of optimising one coalition are repeated in optimising subsequent coalitions. By taking advantage of this recurrence, we can create a dynamic programming (DP) algorithm to calculate the bounded rational Shapley value much more efficiently. Next, we explain this approach in more detail.

To calculate the Shapley value using Algorithm 3, we need to optimise all subsets *C* of the grand coalition one by one. Recall that based on the flexibility heuristic of Eq (10), we initially sort *N* such that Apartment 1 and Apartment *n* are the least and most flexible members, respectively. Then, cOPT independently optimises all apartments using iOPT, and the set of congested time slots given the independent optimisations are identified. If the result of the optimisations (and the congested time slots) up to this point were stored, we could avoid re-computing them 2^*n*^ times. This is because these steps are repeated in each iteration of the collective optimisation.

The next step of cOPT is re-optimising the members of *C* given the congested time slots. This is always done starting from the least flexible member. Clearly, in any subset of *N*, one member is the least flexible, which is exactly the least flexible member in many other subsets as well. Therefore, that member is always re-optimised first in all those coalitions, which always results in exactly the same load profile for that apartment and the same set of congested time slots. For instance, when cOPT wants to optimise any coalition containing Apartment 1, it always performs decongestion starting from Apartment 1, since it is the least flexible member of the coalition. If the re-optimisation of Apartment 1 is successful (i.e., merely its own preferences are satisfied, not necessarily the threshold), the optimisation results and the newly computed congested time slots can be reused in any other coalition of which Apartment 1 is a member. If the re-optimisation is not successful, we revert back to the independently optimised load profile and the corresponding set of congested time slots which were previously stored in the memory. Likewise, when the next least flexible apartment in the coalition is re-optimised the results can be stored in the memory so that they are later used by cOPT in re-optimising other coalitions. Taking advantage of this recursion can greatly reduce the computation needed for optimising the 2^*n*^ subsets of *N*. The following example further illustrates the recursion.

Suppose that in a game of 5 apartments, we want to optimise {1}. We must first optimise all apartments independently, which yields a load profile for all members as well as a set of congested time slots Λ_∅_. If Apartment 1 can be re-optimised such that its temperature preferences are satisfied, a new set of congested time slots, Λ_{1}_, will be yielded. Let us assume that re-optimising Apartments 1 indeed results in satisfying its preferences, but Λ_{1}_ is not empty (i.e., there are some congested time slots). Here, although the best-found cooling plan of Apartment 1 will be reverted to its independently optimised cooling plan (since the threshold is not satisfied), we can store the result of re-optimising Apartment 1 as well as Λ_{1}_ so that they can be reused in optimising any other coalition that contains Apartment 1. Now, suppose that we would like to optimise {1, 2}. After using the stored results from re-optimising {1}, since Λ_{1}_ is not empty, we need to also re-optimise Apartment 2 which will yield Λ_{1,2}_. Regardless of whether Λ_{1,2}_ is empty or not, every time a coalition that contains Apartment 1 and Apartment 2 (e.g., {1, 2, 4, 5}) is optimised, re-optimising Apartment 1 and Apartment 2 will result in the same cooling plans for these two, and the same Λ_{1}_ and Λ_{1,2}_. Therefore, if after each re-optimisation we stored the cooling plans along with the resulting set of congested time slots, we would not need to compute them again in optimising the subsequent coalitions. This way, for each coalition we would need to re-optimise only one apartment, which is essentially the most flexible member. Furthermore, note that when Shapley values are calculated using Algorithm 3, it is important to iterate through the subsets of the grand coalition such that optimising any coalition would depend only on the previously visited ones. To ensure this, we use the natural order of subsets in binary representation. In this representation, a non-empty coalition *C* = {*c*_1_, *c*_2_, …, *c*_*m*_} is represented by the binary equivalent of $2^{c_{1}-1}+2^{c_{2}-1}+\ldots +2^{c_{m}-1}$, where each bit indicates whether or not the corresponding agent is a member of the coalition. For instance, {2, 3} comes immediately before {1, 2, 3} as their corresponding binary numbers are 110 and 111, respectively. Table 3 illustrates the recursion in the collective optimisation using the binary representation. For example, for optimising {1, 2, 4}, the right column shows that the result of optimising {1, 2} is re-used, which is itself optimised using the result of optimising {1}.

**Table 3:** Binary representation of coalitions in the 5 apartments example. The underlined members are the only ones in each coalition that may be re-optimised.

Coalition	A5	A4	A3	A2	A1	From Memory
{}	0	0	0	0	0	−
{1}	0	0	0	0	1	{}
{2}	0	0	0	1	0	{}
{1, 2}	0	0	0	1	1	{1}
{3}	0	0	1	0	0	{}
{1, 3}	0	0	1	0	1	{1}
{2, 3}	0	0	1	1	0	{2}
{1, 2, 3}	0	0	1	1	1	{1, 2}
{4}	0	1	0	0	0	{}
{1, 4}	0	1	0	0	1	{1}
{2, 4}	0	1	0	1	0	{2}
{1, 2, 4}	0	1	0	1	1	{1, 2}
{3, 4}	0	1	1	0	0	{3}
{1, 3, 4}	0	1	1	0	1	{1, 3}
…	…	…	…	…	…	…
{1, 2, 3, 4, 5}	1	1	1	1	1	{1, 2, 3, 4}

Based on the recursion described above, we can construct a DP algorithm to calculate the Shapley value of the apartments more efficiently. This will enable us to (re-)optimise only one apartment per coalition—the most flexible apartment—since the cooling plan of the rest of the members can be used from the previously optimised coalitions. In fact, the time complexity of calculating the Shapley value of all agents can be reduced to *O*(2^*n*^), down from *O*(*n* × 2^*n*^). However, this computational benefit comes at a memory space cost of *O*(|*C*|) for each *C* ⊆ *N*, which implies an exponential space complexity overall. We next formalise the recurrence relation.

Let a coalition sorted in the ascending order of its members’ flexibility (according to Eq 10) be *C* = {1, 2, …, *m*}, such that apartments 1 and *m* are the least and most flexible apartments, respectively. Furthermore, let Λ_*C*_ denote the set of congested time slots obtained by re-optimising *m*, given the set of congested time slots, Λ_*C*∖{*m*}_, obtained by re-optimising the most flexible apartment in *C*∖{*m*} (i.e., *m* − 1). As such, Λ_∅_ is the set of congested time slots after optimising all apartments independently, and Λ_*N*_ is the set of congested time slots obtained by re-optimising apartment *n* given Λ_*N*_∖{*n*}. Moreover, let a vector of re-optimised cooling actions of the most flexible apartment in coalition *C* over an entire day be denoted by $\eta_{m}^{\Lambda_{C\backslash \{m\}}}$, which is obtained by re-optimising *m* given Λ_*C*∖{*m*}_. Note that if *m* cannot be re-optimised based on Λ_*C*∖{*m*}_ such that its temperature preferences can be satisfied, then $\eta_{m}^{\Lambda_{C\backslash \{m\}}}$ will simply be the independently optimised plan that is found by *iOPT*. Denote by $l_{m}^{\Lambda_{C\backslash \{m\}}}$ the vector of best-found cooling load of apartment *m* that is given by Eq (3) using $\eta_{m}^{\Lambda_{C\backslash \{m\}}}$. We can now compute the vector of best-found aggregate cooling load, $l_{C}^{\prime }=\left[l_{C}^{{}^{\prime }1}l_{C}^{{}^{\prime }2}\ldots l_{C}^{{}^{\prime }k}\right]$, of a coalition, *C* ≠ ∅, using the following recursive formula:

$$\begin{array}{c} l_{C}^{\prime }=\{\begin{array}{cc} \sum_{i\in C}l_{i}^{\prime \prime } & \text{if}\;|C|=1\;\text{or}\;\Lambda_{C}\neq \emptyset \\ l_{C\backslash \{m\}}^{\prime }+l_{m}^{\Lambda_{C\backslash \{m\}}} & \text{if}\;\Lambda_{C}=\emptyset \end{array}, \end{array}$$ (14)

where $l_{i}^{\prime \prime }$ is the vector of best-found cooling actions of apartment *i* when it optimises its load independently. Using Eqs (14) and (4) we can easily find the bounded rational value of *C*, i.e., *v*^*BR*^(*C*). Our experiments in the next section verify that the DP method results in a significant reduction of computation time.

## Evaluation of the payments of apartments

We now undertake an evaluation of applying the bounded rational Shapley value to the discount scheme. To this end, we consider a block of 15 apartments participating in the scheme. However, since there are myriad ways in which 15 apartments can require cooling over the course of a day, it is impossible to systematically evaluate their payments with all possible combinations of setpoints, tolerances and comfort periods. Therefore, our experiments investigate how the payments incurred by one apartment change, as its preferences are varied, while other apartments’ preferences remain constant. This is to isolate the effect of each varying parameter on the payments. Our aim is to simulate the preferences and thermal characteristics of the apartments in a way that represents a severe case in terms of satisfying the optimisation constraints–most, if not all, other cases will be arguably easier to deal with. One such case is realised when a maximum peak occurs in all congested time slots. The following preferences and thermal characteristics ensure this.

**Fig 5:**
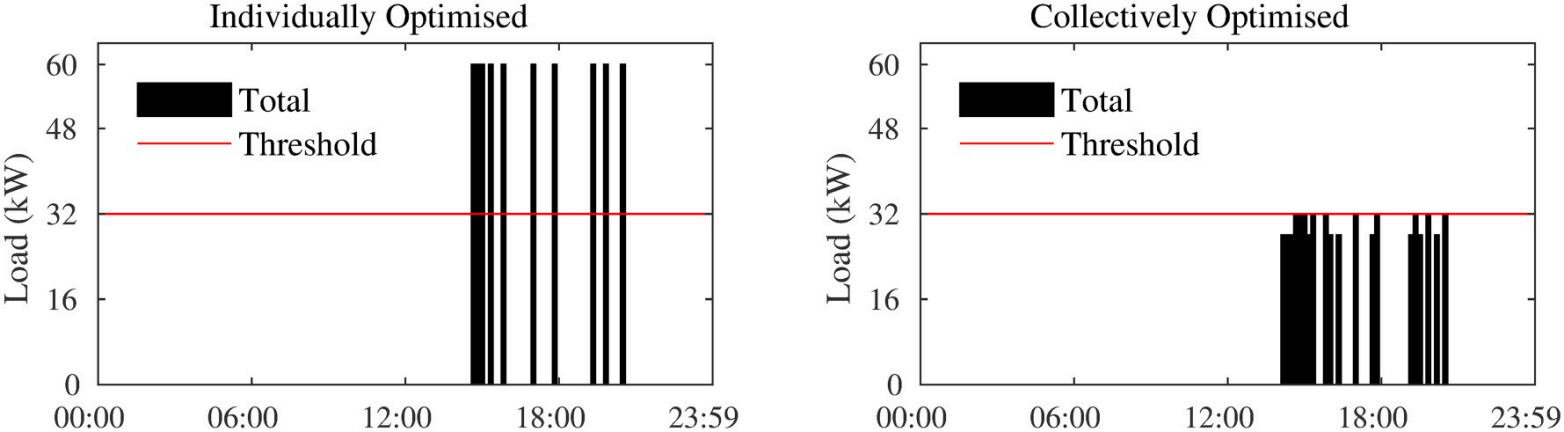
Total load when apartments optimise individually and collectively.

We assume that the AC system in all apartments operate on a 10-minute cycle, i.e., a cooling decision is made for each 10-minute interval in a day (Δ*t* = 1/6hr), and thus, *K* = [1, …, 144]. We also assume that the AC systems in all apartments are similar, with an *r* value of 1.0 °*C*/*h* and consume at a rate of 4.0 kW when on (i.e., *P*_*i*_ = 4.0 kW). Therefore, the maximum possible load of the entire block is 60 kW. When the apartments participate in the scheme, a threshold (*ψ*) at 32 kW (representing a 47% reduction), is set on the block’s load. If the aggregate load throughout the day is always below 32 kW, the price per kWh of energy consumed over the entire day is $0.08, otherwise it is $0.15. All apartments have the same setpoint of 22°*C*, tolerance level of 1°*C*, and require cooling from 15:00 to 21:30. As can be seen in Fig 5, these assumptions ensure that when all apartments optimise their load independently, their AC is run during exactly the same time slots, causing a maximum peak (i.e., 60 kW) in all congested time slots. Moreover, to exclude the impact of thermal characteristics of the other 14 apartments, we assume that all leakage rates are equal and as follows: *α* = 0.005, *β* = 0.005, and *γ* = 0.05. These numbers are based on realistic values learned from real data collected from a number of apartments in Jeddah, Saudi Arabia.

The total cooling load when the 15 apartments, with the above settings and parameters, optimise their load individually and collectively are shown in Fig 5. As is evident, using cOPT (Algorithm 2), the apartments are able to find an alternative cooling plan such that in addition to their individual temperature preferences, the threshold of 32 kW is also met.

Having established the satisfiability of the threshold constraint, we now vary the preferences of one of the 15 apartments, and keep the preferences and thermal properties of others constant. We compare the payments that the apartment can be charged for its consumption in four different cases: (i) when the apartment does not sign up to the discount scheme and optimises its load independently, (ii) when the apartment optimises its load as a member of the coalition and benefits from the discount, but only its consumption in the grand coalition is considered (unlike the Shapley value which considers all subsets of the grand coalition), (iii) when the apartment optimises its load as a member of the coalition and each apartment receives an equal share of the total saving from the discount (the difference between the payment of the grand coalition at the discounted and normal rates is equally divided and deducted from the payment of each apartment), and (iv) when the apartment optimises its load as a member of the coalition and receives its bounded rational Shapley value. We additionally show the bounded rational Shapley value of the rest of the apartments, which are essentially equal due to identical settings. The value of a coalition in this experiment is calculated as per Eq (5), which represents its bounded rationality value.

**Fig 6:**
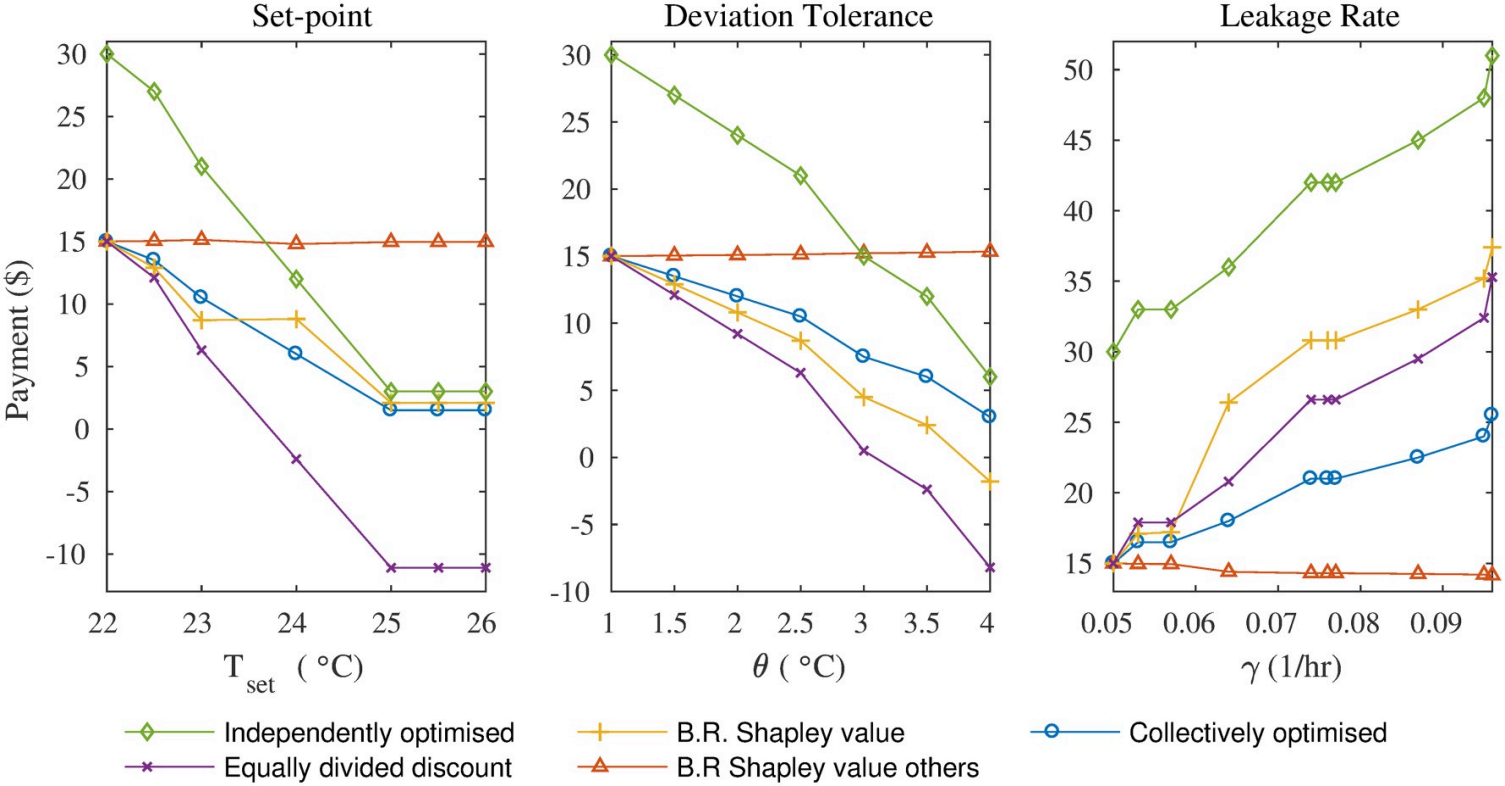
The payments of an apartment versus setpoint, deviation tolerance and leakage rate.

In our experiments, we investigate the relationship between payments and different values of setpoint temperature, setpoint deviation tolerance, and leakage rate, the results of which are depicted in Fig 6. These comparisons enable us to develop an in-depth understanding of how sensitive the bounded rational Shapley value and the other naive payment mechanisms are to the variations in the aforementioned parameters. As expected, all three experiments verify that the payments based on the bounded rationality Shapley value are consistently smaller than what the apartment would have to pay if it did not participate in the scheme.

To explore the relationship between the setpoint temperature settings and payments, we vary *T*_*set*_ from 22°C to 26°C, while leaving the other preferences unchanged. We calculate the four payment cases described above for each setpoint temperature setting, and calculate the corresponding payment that the apartment incurs for a single day of cooling. From Fig 6, it is evident that the relationship between the setpoint temperature and payments follows our intuition that when the setpoint is increased, less cooling is required and hence the payment reduces. This reduction occurs until the setpoint is set so high that very little cooling is needed, at which point the payments do not change. It is also evident that an apartment stands to benefit from its participation in the scheme by receiving its bounded rational Shapley value, since the payment it incurs for a setpoint setting is always lower than what it would incur if it chose to optimise its cooling load independently. Somewhat unsurprisingly, the bounded rationality Shapley value of other apartments across different setpoints of the apartment in question remains equal but consistently higher. This shows that the extra flexibility of this apartment (due to its higher setpoint) does not make other members better off or worse off. However, higher setpoint results in less cooling, hence the lower payment compared to others. Moreover, when the independently optimised and the bounded rationality Shapley value curves converge, it means that the energy consumption in and out of the coalition are very close, which occurs when the setpoint is set relatively high. Note that if the payment goes below zero–which could possibly occur due to the proportion of discount and consumption–the equivalent credit can be awarded to the apartments so that it can be used towards their bill. Furthermore, note that when the setpoint is 22.0°C, all payments (including those of other apartments) except the independently optimised one are equal. This indicates that when all apartments have identical settings, all methods result in the same payment. Therefore, in that case one can simply use the equally divided discount method whose computation is much simpler than the bounded rational Shapley value.

Previously, we described how *θ* is indicative of the sensitivity of an apartment to deviations of the internal temperature from the setpoint. We next explore how the payment incurred for a particular setting changes as *θ* is varied from 1.0°C to 4.0°C. Again, we calculate the corresponding payment cases for each setting, for a single day of cooling. From Fig 6, it is evident that the relationship between *θ* and payments is almost linear. As *θ* is increased, the payments decrease, which shows that the amount of energy required to satisfy the setpoint constraint becomes less and less. Therefore, an apartment with a high setpoint tolerance is easier to satisfy, which is one of the facts that we exploit in the collective optimisation to satisfy the load threshold. Observe that similar to the setpoint experiment, the payments of the other apartments are constant throughout.

In previous sections, we established how the leakage rate, *γ* is related to the level of insulation of an apartment. We also mentioned how an apartment with a high *γ* value will typically incur higher energy consumption. Following this trend, we now establish the relationship between *γ* and the payment that one apartment incurs for a single day of cooling. To do so, we vary *γ* from 0.05°C/hr, representing a relatively high level of insulation to to 0.096°C/hr, representing a relatively poorly insulated apartment. For each *γ* value, the payment that the apartment incurs for a single day of cooling is then calculated based on the four payment cases. Fig 6 depicts that the relationship between the leakage rates and the payments is monotonically increasing in all cases. The discounted payments are initially close, but as the apartment becomes leakier, the payments diverge, which makes it more justifiable to choose the method that is fair. Of the three parameters considered in these experiments, the payments seem to exhibit more sensitivity to leakage rate, as small changes in *γ* results in relatively higher changes in the payments. Also, the larger gap between the bounded rationality Shapley value and the independently optimised curves indicates a higher sensitivity of the payments to thermal leakage when the apartment is in the coalition than when it is not. This can further motivate a leaky apartment to sign up to the scheme and benefit from the better insulation of other apartments which require less cooling. Interestingly, the other apartments also stand to benefit from the existence of a leakier apartment in the coalition. As can be seen in the plot, the bounded rationality Shapley value of other apartments (whose leakage is less) slightly decreases with higher values of *γ*. This is in contrast to the two previous experiments.

**Fig 7:**
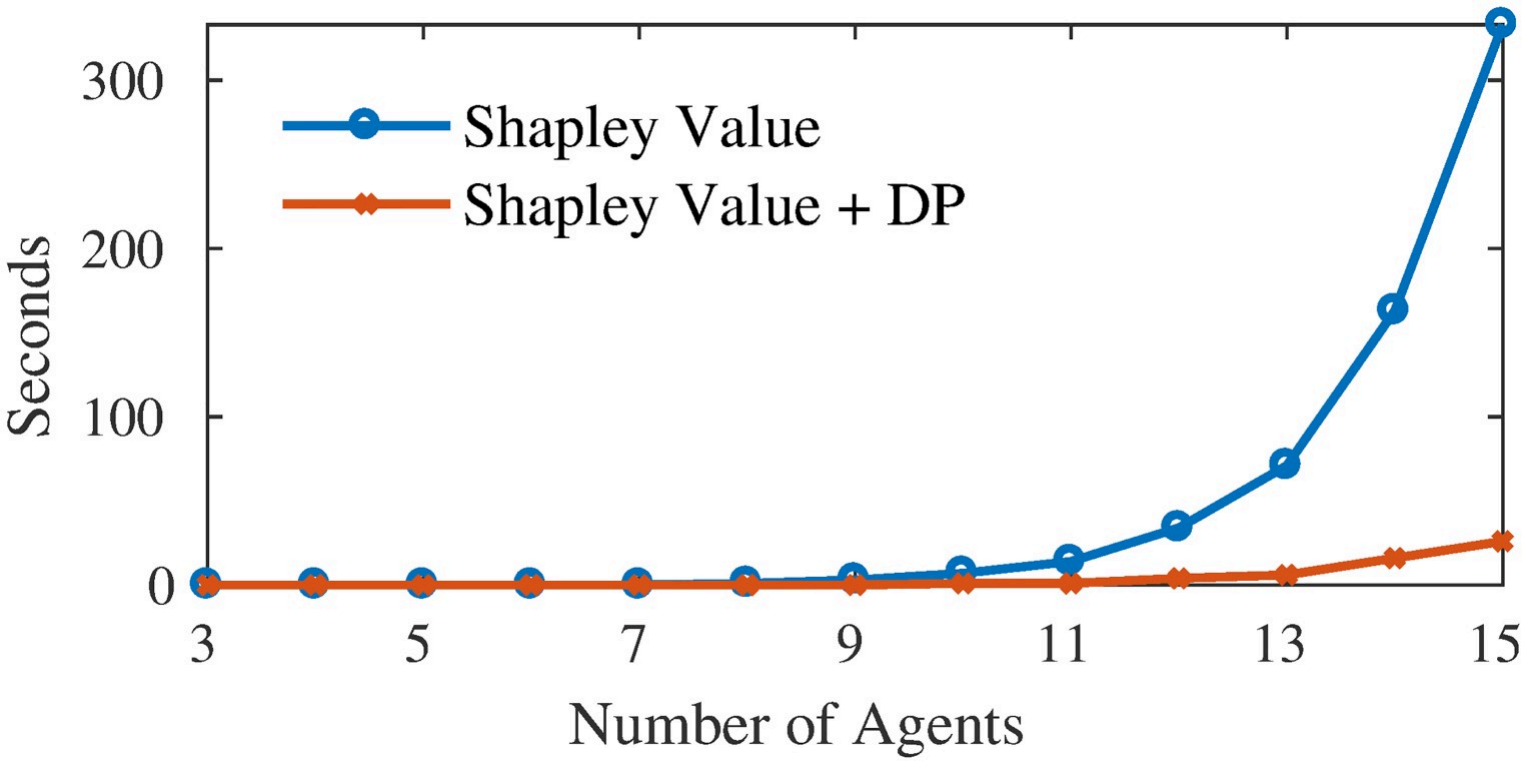
Computation time of the Shapley value with different coalition sizes.

The three experiments discussed above demonstrate the interesting properties of the bounded rationality Shapley value as a payment mechanism. We also note the computational advantage of using the bounded rational Shapley value particularly when it is calculated using the DP method described in subsection efficient calculation of the Shapley value using dynamic programing, which significantly reduces computation time. Fig 7 shows a comparison of the time it takes to calculate the bounded rational Shapley value of an entire block, when there are 3 to 15 apartments, using the DP method and without it. In both cases, the Shapley value is calculated using Algorithm 3, which is itself a significantly more efficient implementation of the standard Shapley value formula. It is evident that an exponential gain in computation time is achieved with the DP method.

## Conclusions and future work

In this article, we considered a real-world problem where a block of apartments participate in a demand response program to ensure their aggregate cooling load does not exceed a certain threshold. In return, the apartments receive a discount for coordinating their loads. In this problem, a coalition of apartments needs to optimise its members’ use of air conditioning subject to the individual temperature preferences of each apartment and the given threshold. Due to the magnitude of the constraints involved, computing the optimal load of a coalition is computationally intensive. Instead of solving this problem optimally, we used a greedy algorithm which produced suboptimal solutions at a higher speed. Consequently, a suboptimal value for each coalition could be found, enabling us to calculate the cost of coalitions in a reasonable time. However, calculating the Shapley value in this setting still entails an extra computational challenge, namely solving an exponential number of optimisation problems. Since optimising the load of coalitions with respect to all constraints can take a considerable amount of time, and the agents do not have infinite computational resources, the agents are considered to be computationally bounded rational. While using the Shapley value as a fair division of the optimal value of the grand coalition may not be possible in practice, we proposed that based on the additivity axiom of the Shapley value it is still possible to obtain a fair division using the aforementioned greedy algorithm, which is fast but not necessarily optimal.

The Shapley value given the suboptimal coalition values (which we call the bounded rational Shapley value), is not only easier to calculate, but also provides a division of the grand coalition that is fair in the following sense: all agents are rewarded for their contribution to the total cost of the block (at the discounted rate), and simultaneously penalised for their contribution to the extra cost that is due to the discrepancy between the suboptimal and optimal solutions.

Since in many real world problems the number of agents is beyond what the Shapley value can be easily calculated for, one of our concerns is the scalability of the bounded rational Shapley value. Therefore, in future work, we would like to explore how our approach can be applied to similar demand response scenarios consisting of large number of agents. We would be interested in investigating how approximation techniques could be used to scale up the game, without losing the fairness properties of the bounded rational Shapley value.

## Supporting information

S1 Dataset The temperature data and thermal parameters of individual apartments. (ZIP)
